# Evaluation of the Propensity of Niobium to Absorb Hydrogen During Fabrication of Superconducting Radio Frequency Cavities for Particle Accelerators

**DOI:** 10.6028/jres.115.025

**Published:** 2010-10-01

**Authors:** R. E. Ricker, G. R. Myneni

**Affiliations:** Materials Science and Engineering Laboratory, and Technology, Gaithersburg, MD 20899-8553; Thomas Jefferson National Accelerator Facility, Newport News, VA 23606

**Keywords:** corrosion, diffusion, electropolishing, fabrication, hydrogen absorption, niobium, particle accelerator cavities, superconducting radio frequency

## Abstract

During the fabrication of niobium superconducting radio frequency (SRF) particle accelerator cavities procedures are used that chemically or mechanically remove the passivating surface film of niobium pentoxide (Nb_2_O_5_). Removal of this film will expose the underlying niobium metal and allow it to react with the processing environment. If these reactions produce hydrogen at sufficient concentrations and rates, then hydrogen will be absorbed and diffuse into the metal. High hydrogen activities could result in supersaturation and the nucleation of hydride phases. If the metal repassivates at the conclusion of the processing step and the passive film blocks hydrogen egress, then the absorbed hydrogen or hydrides could be retained and alter the performance of the metal during subsequent processing steps or in-service. This report examines the feasibility of this hypothesis by first identifying the postulated events, conditions, and reactions and then determining if each is consistent with accepted scientific principles, literature, and data. Established precedent for similar events in other systems was found in the scientific literature and thermodynamic analysis found that the postulated reactions were not only energetically favorable, but produced large driving forces. The hydrogen activity or fugacity required for the reactions to be at equilibrium was determined to indicate the propensity for hydrogen evolution, absorption, and hydride nucleation. The influence of processing conditions and kinetics on the proximity of hydrogen surface coverage to these theoretical values is discussed. This examination found that the hypothesis of hydrogen absorption during SRF processing is consistent with published scientific literature and thermodynamic principles.

## 1. Introduction

The propensity of active metals to absorb hydrogen during service in environments containing water or water vapor is a well-known and thoroughly studied phenomenon [3–5], but the possibility of similar processes occurring during fabrication is frequently overlooked. During the fabrication of niobium (Nb) superconducting radio frequency (SRF) cavities for use in particle accelerators, there are a number of processing steps where the passivating film of niobium pentoxide (Nb_2_O_5_) is removed by chemical or mechanical means. When this occurs, the underlying metal is put into direct contact with the processing environment and is free to react with this environment. If the metal is sufficiently active, then the subsequent reactions may produce hydrogen at activities that may result in the metal absorbing hydrogen and in some cases, the hydrogen activity may be sufficient to supersaturate the metal with respect to hydride phases. When the passive film reforms, this film may block egress of the absorbed hydrogen allowing it to remain in the metal and influence performance during subsequent processing steps or inhibit the ability of a part to perform its intended function in-service.^1^

Of particular concern for Nb SRF cavities is the sharp increase in RF losses at critical magnetic fields that limits the operational accelerating gradient of Nb SRF cavities [6–8]. This phenomena is known as the “high field Q-slope” or “Q-drop” in the SRF literature. One potential explanation for this phenomenon is that the hydrogen absorbed during processing is responsible and that variability with processing conditions due to variation in the hydrogen content or distribution. The effects of processing variables and post-processing treatments on the “Q-drop” phenomena have become the subject of research at a number of institutions [9–12] and Coviati et al., [8] recently reviewed this work. Since the hypothesis that hydrogen is responsible for the “Q-drop” phenomena will not gain the widespread acceptance needed for open debate until the hydrogen sources are clearly identified and their relative influences quantified, the objective of this paper is to identify potential hydrogen sources in the processing environment used for the fabrication of SRF cavities and systematically evaluate the relative magnitudes of the hydrogen activities the different environments can produce based on thermodynamic driving forces and established scientific principles.

## 2. Background

### A. Niobium Metallurgy

Niobium (Nb) is the 41’st element of the periodic chart and has a relative molecular mass of 92.906, a specific gravity of 8.4. Niobium is also known as Columbium (Cb) in the older literature. Niobium is steel-grey body centered cubic (bcc) metal that has found a number of uses in aerospace and medical applications [13, 14]. However, the vast majority of Nb reduced from raw ores each year is used as an alloying element in steels [13, 15]. Niobium has a positive influence on the strength and formability of steel alloys and is used as a microalloying element in the ultra low carbon steels or interstitial free steels used for forming automotive bodies [16–18] and in the high-strength low alloy steels used in shipbuilding [19–21] and the new X-series of alloys used for pipeline construction [22–24]. These alloys are noted for their formability, strength, and resistance to hydrogen embrittlement though hydrogen embrittlement in these steels has been attributed by some authors to the affinity of Nb rich phases for hydrogen [25]. Niobium also finds many uses in the form of niobium oxide (Nb_2_O_5_) due to the stability, clarity, and index of refraction of glasses made with this oxide [26]. One of the properties that Nb is best known for is superconductivity [26]. Niobium is a classical (low temperature) superconductor that transitions from normal metallic conduction to superconductivity at 9.2 K making it ideally suited for the construction of superconducting radio frequency (SRF) cavities for high-energy physics experiments where a project may require over 5.0 × 10^5^ kg of ultra high purity metal [27].

Since understanding the forming properties of this element would help us understand fundamental issues in forming and the influence of this element on the formability of steels, the Metallurgy Division of NIST began working with Department of Energy, Thomas Jefferson National Accelerator Facility (JLab). Initially, the NIST Chemical Science and Technology Laboratory (CSTL) and the NIST Center for Neutron Research (NCNR) collaborated with JLab investigating techniques for analyzing the hydrogen content of processed Nb sheet [28, 29]. The Metallurgy Division of the Materials Science and Engineering Laboratory (MSEL) became involved when issues related to residual stresses in deep drawn cavities and springback became a concern [30]. These interactions resulted in cross-fertilization of research ideas including experiments on large grained samples [31], the use of mechanical properties to detect interstitial hydrogen [32], and the hypothesis of hydrogen absorption [5].

### B. Niobium is an Active Metal That Passivates

Noble and active are terms commonly used to classify the behavior of metals. In general, the term noble is applied to metals that are immune to reaction with their environment and active metals spontaneously react with their environment. Clearly, this depends on the environment. From an aqueous corrosion standpoint, an active metal is one that will spontaneously react in normal aqueous environments under ambient conditions without the application of any external force or energy. In scientific terms, an active metal is one where the metallic state is not the lowest energy state for the atoms of the element in contact with water molecules. Instead, the atoms of an active metal prefer to exist as positively charged ions in either a soluble complex or in an oxide, hydroxide, or similar compounds. A metal is said to be noble if the metallic state is the lowest energy state in the environment and the metal is immune to reactions unless an external force is applied. Ions of noble metals in these environments will spontaneously reduce their oxidation states and plate out on surfaces in the metallic state if species that can be oxidized are present (displacement plating).

**Fig. 1:**
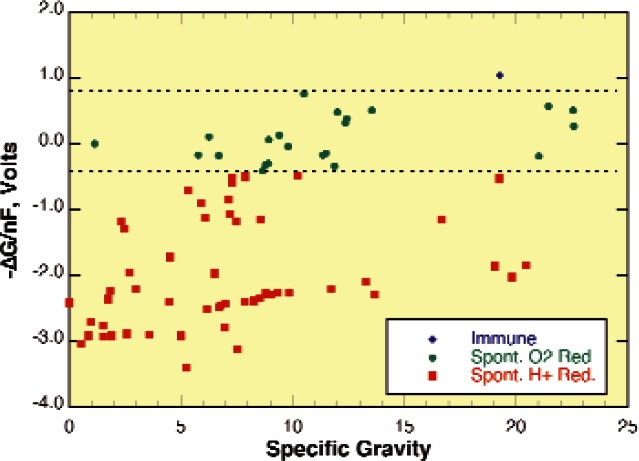
Equilibrium potentials for pure metals at pH 7.00 as a function of density [33].

Figure 1 is a plot of the specific gravity (density) of the metallic elements of the periodic chart and the Gibbs free energy for the reaction of these elements with water to form an oxide or hydroxide in neutral aqueous solutions [33]. There are two dashed lines in this figure that identify the potentials where the cathodic reduction reactions

(1) $$\frac{1}{2}O_{2}(diss)+H_{2}O(l)+2e^{-}\to 2OH^{-}(aq)$$

and

(2) $$H_{2}O(l)+e^{-}\to OH^{-}(aq)+\frac{1}{2}H_{2}(g)$$

are at equilibrium under ambient conditions in neutral pH water (pH ≈ 7.0). All of the elements with equilibrium potentials below the upper dashed line in this figure produce electrons with enough energy when they oxidize to spontaneously reduce any oxygen dissolved in aqueous solutions according to the reaction of Eq. (1). The second, lower, dashed line is a similar line for the cathodic reduction reaction of Eq. (2). Metals that have equilibrium potentials below this line produce electrons with enough energy when they oxidize to spontaneously reduce the hydrogen ions in water. That is, they will drive the reaction of Eq. (2) in the forward direction and the rate will vary with the excess Gibbs energy or distance below this line. Metals below the lower line spontaneously react with any aqueous environment and metals between the two lines react in aqueous environments only when O_2_ is present. Metals above the upper line require more aggressive oxidizers to make them react in this environment.

The discussion above only examined reactions with neutral *pH* water. There are a wide range of *pHs* and aeration levels consistent with the term “aqueous environments under ambient conditions” and some metals will prefer the metallic state in some conditions and ionic states in others. To help understand these cases, Pourbaix and co-workers [34] advocated the use of electrochemical equilibrium diagrams (*E-pH* diagrams). For these diagrams, the solution *pH* is plotted on the horizontal axis with the driving force for electrochemical reactions on the vertical scale. At constant pressure, the force driving chemical reactions is normally quantified by the thermodynamic quantity Gibbs free energy or Gibbs energy (*ΔG*). For reactions on the surface of an electrode immersed in an electrolyte, the driving force is quantified by the potential difference between the electrode and a standard reference reaction with a defined or well-known Gibbs free energy and reference potential. The hydrogen evolution reaction at standard state is frequently used for this reference potential and potentials measured against this electrode (*E_SHE_*) are related to Gibbs free energy through the relationship

(3) $$E_{SHE}=-\Delta G/nF$$

where *n* is the change in the oxidation state, *F* is Faraday’s constant, and *ΔG* for the hydrogen evolution reaction at standard state is defined as zero by convention of the International Union of Pure and Applied Chemists (IUPAC) [35]. This is a very important relationship because it relates electrochemical measurements of voltage and current directly to chemical thermodynamics and kinetics. It also creates a direct link between these quantities and the activity of hydrogen on the surface of an electrode. That is, since the chemical reaction occurring in the standard hydrogen electrode that is used as the zero point of potential for electrochemical measurements is

(4) $$H^{+}+e^{-}\to \frac{1}{2}H_{2}(g)$$

and hydrogen is a reference phase for thermodynamic tables with *ΔG* = 0 for all reactants in their standard states [35], electrode potentials measured against this scale are directly relatable to chemical thermodynamics. Furthermore, since the standard state for this reaction is hydrogen ions with an activity of 1.0 (pH = 0) on the surface of platinum metal in equilibrium with hydrogen gas at a pressure of 10^5^ Pa (0.987 atm), this relationship also directly relates tabulated data on chemical thermodynamics and the Gibbs free energy change for a reaction (*ΔG*) to a potential and the activity of hydrogen molecules on the surface of an electrode where that reaction is occurring. That is, potential, Gibbs free energy, and the activity of hydrogen molecules on the surface of the electrode (fugacity) are all related through the reaction of Eq. (4), the relationship of Eq. (3), and the standard principles of chemical thermodynamics.

**Fig. 2:**
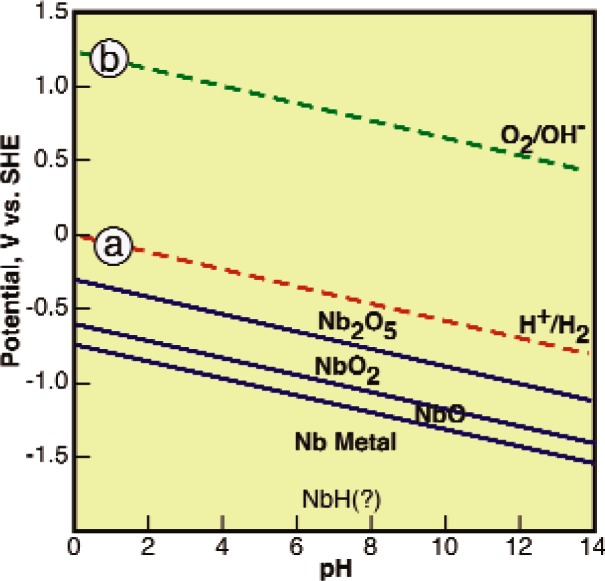
Electrochemical equilibrium (E-pH) diagram for Nb in water without consideration of niobium hydride phases.

Figure 2 is the *E-pH* diagram for Nb in water determined using the thermodynamic data of Wagman [35] and Pourbaix [34, 36]. This figure contains two dashed lines labeled “a” and “b” to represent the reactions of Eqs. (1) and (2) respectively similar to the lines in Fig. 1, but now with a slope of −0.059 V/pH since the equilibrium potential for these reactions depends on the pH of the solution. By examining this figure, it can be seen that Nb has 4 phase fields (Nb, NbO, NbO_2_, and Nb_2_O_5_) and no soluble ions. In the range between the dashed lines where water is stable, the lowest energy state for Nb atoms is as ions in the pentoxide. This diagram shows that Nb has a very high reactivity with water, but also has high probability that it will react to form a passivating surface oxide film that makes it appear inert. The high reactivity is shown by the potential difference between the upper edge of the field where metallic Nb is stable and the line where the fugacity of hydrogen is 10^5^ Pa (line “a”). This indicates a large driving force for the reduction of hydrogen ions and the lack of any soluble ions, assuming the fluoride ion or other ions that form soluble complexes are not present, indicates that mass transport will not be able to remove the products of this reaction before the surface becomes covered isolating the underlying metal from the environment. In other words, Nb is an active metal that will appear to be noble due to the formation of a passivating layer (i.e., a passive metal, or in the older literature, a valve metal) [37]. In some cases, the underlying metal is so active that the even the application of strong cathodic currents cannot reduce the protective oxide film.

Figure 2 was calculated considering only Nb metal, ions, and oxide or hydroxide phases. This diagram neglects the possibility of the formation of Nb hydrides and “NbH(?)” is included in the figure to indicate this fact. The purpose of this figure is to establish the phases that may be present on Nb at the start of processing. Then, the reactions between these phases and the processing environment will be considered with the reaction products and their activities estimated. Once the activities of the reaction products are estimated, then the effects of the presence of these products at the expected activities will be considered including the possibility of the nucleation of niobium hydride phases.

### C. Passive Metals and Hydrogen Absorption

The ability of active metals to react with water and absorb hydrogen is a well-known phenomenon [3–5]. It is also well known that similar reactions can result in hydrogen uptake during chemical processing, finishing, or cleaning of metals [38]. However, it is not uncommon for these possibilities to be ignored for a number of reasons including: (i) under appreciation of the activity of the metals involved (passivity creates a false impression of nobility), (ii) limited exposures times, (iii) ambient or low temperatures, and (iv) the assumption that absorbed hydrogen will desorb before it can cause harm. There are standardized tests developed specifically for the evaluation the effects of cleaning and processing solutions on hydrogen uptake by metals [39]. In the case of passive metals, the passive films can be a very effective barrier to hydrogen uptake due to the relatively low solubility and diffusivity of hydrogen in these films as well as their effect on reaction kinetics and hydrogen reduction rates. Unfortunately, the low solubility and diffusivity of these films can also block hydrogen desorption. Frequently, steels are plated with Cu or Cd to prevent hydrogen absorption and it has been found that if the samples are not hydrogen free before they are plated then hydrogen embrittlement may occur. Plating and passivation have been used to seal hydrogen into samples and prevent hydrogen desorption between charging experiments and hydrogen content measurements.

**Fig. 3:**
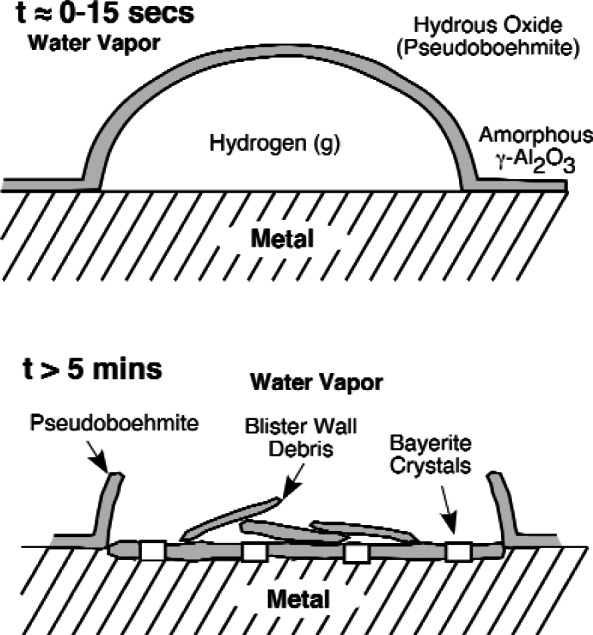
Schematic of blisters observed when an Al alloy was exposed to water vapor in an electron microscope [1].

**Fig. 4:**
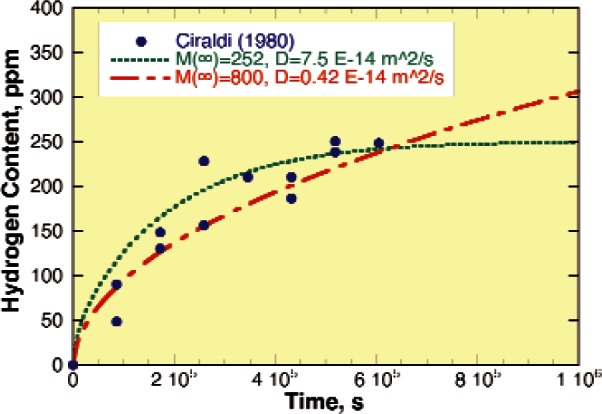
Hydrogen content of an Al alloy measured after abrasion for different times in aqueous slurry and predicted absorption rates for different hydrogen solubility and diffusion coefficients [2].

Charging hydrogen into passive metals to study its effects on properties can be particularly difficult for metals that form oxide films that cannot be reduced by strong cathodic currents. Typically, four different approaches are used when exposure to hydrogen gas and cathodic charging fail to introduce hydrogen into these metals: (1) cathodic charging in aggressive solutions containing species that form soluble complexes (e.g., F^−^), (2) removal of the passive film and replacing it with a coating of Pd or Ni [40], (3) exposure to water vapor at moderately elevated temperatures [41], and (4) mechanical abrasion [2]. All have proven successful for different metals and alloys, but we will focus on those relevant to hydrogen uptake during Nb processing. Figure 3 shows a schematic for the hydrogen absorption mechanism observed by Scamans and Tuck [1] in samples of an Al alloy in a transmission electron microscope when water vapor was allowed to leak into the vacuum chamber. The passive film was observed to form hydrogen gas blisters that cracked allowing the environment access to the underlying metal. Exposure to water vapor saturated air is a common test for Al alloys and similar blisters are frequent observed [42]. Slow continuous abrasion has also been used on Al alloys [2]. Figure 4 plots hydrogen content data as a function of polishing time taken from the work of Ciraldi [2]. This figure clearly shows a slowly increasing hydrogen content with time, but one that agrees well with the diffusivity data for hydrogen in Al alloys available in the literature [5]. In some cases, hydrogen absorption by a passive metal is so difficult when the passive film is stable that many do not realize that these metals are susceptible to the influence of hydrogen, but unique processing conditions that are specifically designed to attack or remove the passive film defeats this natural protection system enabling hydrogen entry and embrittlement.

### D. Hydrogen Absorption and Evolution

**Fig. 5:**
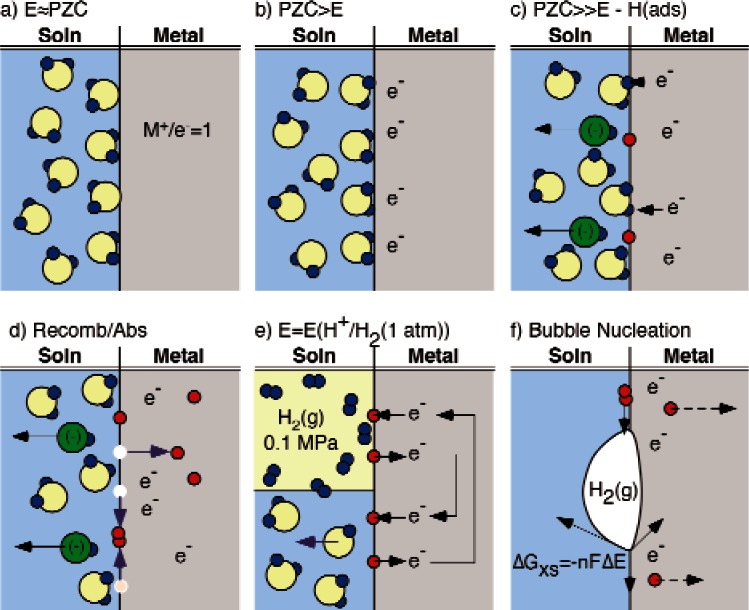
Schematic illustrating the steps in cathodic reduction and hydrogen evolution on the bare surface of a metal.

Figure 5 is a schematic representation of the steps in hydrogen evolution that accompanies anodic dissolution on the bare surface of an active metal in an aqueous solution. This sequence begins with the metal in the solution at the potential of zero charge (PZC) in Fig. 5(a). This is a theoretical point where the adsorbed polar water molecules are randomly oriented on the surface of the electrode. At any other potential, the adsorbed molecules will have a preferred orientation. In the case of a negatively charged active metal such as Nb, the water molecules will become oriented with the positive hydrogen cations adsorbed down against the surface of the metal as shown in Fig. 5(b). If the potential gradient is sufficient, electrons will tunnel through the space charge layer reducing the hydrogen cations creating adsorbed H atoms and hydroxyl ions (Fig. 5(c)). This results in the production of adsorbed hydrogen atoms on the surface the activity of which is determined by the potential gradient or the chemical potential. These adsorbed hydrogen atoms (H(ads)) will do one of two things: (i) diffuse across the surface until they bump into another adsorbed hydrogen atom and recombine to form an adsorbed hydrogen molecule as in the reaction

(5) $$H(ads)+H(ads)\to H_{2}(ads)$$

or (ii) instead of hopping in the plane of the surface the adsorbed H atoms can jump into the surface where they become absorbed H atoms according to the reaction

(6) H(ads)→H(abs).

It is important to recognize that these are not sequential reactions, but are parallel paths that are both taken and are both driven by the concentration of the adsorbed hydrogen atoms on the surface. At some point, the concentration of the adsorbed H_2_ molecules on the surface is the same as that which would exist for equilibrium with the solution saturated with H_2_(g) at 1 bar pressure (10^5^ Pa). The potential for this equilibrium, Fig. 5(e) is known as the hydrogen evolution potential even though hydrogen is not evolved at this potential. If the potential is reduced further, the fugacity of the hydrogen molecules on the surface continues to increase until there is sufficient driving force to nucleate bubbles of hydrogen gas on the surface as illustrated in Fig. 5(f). The pressure of hydrogen required to nucleate these bubbles is much greater than atmospheric pressure, so once nucleated, these bubbles rapidly expand until the pressure in them approaches atmospheric pressure and the bubbles appear to “pop” on the surface.

The purpose of this discussion of the detailed steps in the process of hydrogen evolution and absorptions was to make four points:

1. *The activity of the absorbed hydrogen is not limited to the external pressure.*—The hydrogen is generated as adsorbed atoms on the surface, but this hydrogen cannot leave the surface until it recombines with other hydrogen atoms to form molecular hydrogen and then enough molecules aggregate to enable heterogeneous nucleation of gas bubbles. The fugacity of the adsorbed hydrogen atoms must exceed the external pressure to provide enough energy to drive the kinetics of these processes. This fugacity can be orders of magnitude greater than the external pressure [43–45].
2. *Hydrogen can be absorbed when bubbles are not observed.*–Since a surface concentration corresponding to a pressure much greater than ambient pressure is required to drive the kinetics hydrogen evolution, hydrogen can be absorbed into metals even when hydrogen gas bubbles are not observed.
3. *Promoting hydrogen evolution (bubbling) can reduce absorption.*–Alterations to the alloy or environment that reduce the overpressure required to drive recombination and nucleation will accelerate bubbling, but will reduce the hydrogen surface concentration driving absorption [44, 46]. Conversely, alterations that inhibit the processes of hydrogen evolution will tend to increase the fugacity of hydrogen on the surface.
4. *Hydrogen reduction comes first.*—Fig 2 shows that Nb wants to passivate by the formation of an oxide when it comes into contact with water, but Fig. 5 shows that the polar water molecule will tend to be adsorbed with the hydrogen anion between these two reactants. This physical arrangement, and the assumption that electron tunneling will be rapid compared to the rearrangement of atoms required for oxide formation and growth, implies that the Nb atoms will give their electrons to the hydrogen first and then bond with the oxygen.

A common misconception is that the external air pressure is the maximum surface fugacity of hydrogen that can be produced by electrochemical reactions, because it is the pressure in the hydrogen bubbles on the surface. A better scenario for understanding this kinetic limit to the fugacity of hydrogen on the surface is to think of it as the pressure required to generate the observed bubble nucleation rate in a sample that was instantly transferred from a high pressure gas chamber to the solution.

## 3. Analysis of Reactions

These calculations assume that the passivating film on Nb has been removed by chemical or mechanical means and that the underlying metal has been exposed to the unaltered processing environment. That is, the calculations will be based on direct reaction between Nb metal and the nominal or bulk chemistry of the processing environment. The relative propensity for hydrogen absorption will be quantified by the calculation of the activity of hydrogen on the surface required for the postulated metal-environment reaction to be in equilibrium. This activity of hydrogen will be expressed in terms of the equivalent pressure or fugacity required for equilibrium. The standard values used for the calculation of hydrogen fugacity are based on measurements of equilibrium for hydrogen at different pressures on platinized Pt, and strictly speaking are true only on for this surface, but Valand et al., [47] compared different metals and concluded that there was no significant difference between the metals examined (Ni, Fe, Cu, Ag, and Au). The sequence of events considered in this report is as follows:

1. Nb metal covered with a passivating layer of Nb_2_O_5_ as indicated by the region between the dashed lines in Fig. 2 is placed in the processing environment.
2. Processing commences and passive film breakdown or rupture events occur. These events may be due to chemical or mechanical processes, but they probably occur at small, occluded, sites distributed over the surface. The actual size, distribution, repassivation rate, and frequency of these events will depend on the particular processing conditions.
3. Each passive film rupture or breakdown event results in sudden contact between three phases: (i) the metal, (ii) remnants of the surface oxide, and (iii) the processing environment. At the moment these three phases come into contact, the composition of each phase is essentially that of the bulk of the phase.
4. Reactions begin immediately and since mass transport is not required initially, reaction rates are determined by the driving forces for the initial (bulk) concentrations of the reactants and the activation energies for the reactions.
5. Reactants are consumed in the occluded region of the passive film breakdown and reaction products accumulate. Mass transport is required to replenish reactants and remove products and this slows reaction rates and locally alters the chemical environment (polarization). The accumulation of reaction products in the occluded region of the passive film breakdown promotes the reformation of the passivating film and repassivation of the breakdown region probably occurs for Nb in aqueous solutions due to the large potential difference (≈ 0.75 V) between the region where water is stable and Nb metal is stable.
6. The whole process is repeated such that at steady state passive film rupture and repair events are continuously occurring at small, localized, regions distributed over the surface of the sample with the rate, size, and distribution of these events determined by the chemical and mechanical conditions of the process. In some cases, repassivation may occur so quickly that the time bare metal is actually in contact with the solution may be less than a millisecond as found for aluminum alloys [48].

### A. Reactions With Water and Dilute Aqueous Solutions ([H_2_O] ≈ 1)

In water and in many solutions where the activity of water can be assumed to be unity, the reactions between Nb and water can be summarized as

(7) $$xNb(s)+yH_{2}O(l)↔Nb_{x}O_{y}(s)+yH_{2}(g)$$

where *x* = 1 or 2 and *y* = 1, 2, or 5 depending on the stoichiometry of the oxide being formed. Keeping in mind the discussion of Sec. 2, this reaction is a summary of the starting and ending points of the process with Gibbs function (*ΔG*) for the free energy change in the reaction given by the relationship

(8) ΔG=ΔG°+RTlnK

where *ΔG*° is the free energy change for all reactants in their standard states and *K* is the reaction constant

(9) $$K=\frac{\prod [\text{Products]}}{\prod [\text{Reactants}]}$$

where the brackets imply the activity of the species within them. Combining Eqs. (3)–(5) and assuming the *ΔG* = 0 at equilibrium gives (7)–(9)

(10) $$K=\frac{\left[Nb_{x}O_{y}\right]{\left[P\left(H_{2}\right)\right]}^{y}}{{\left[Nb\right]}^{x}{\left[H_{2}O\right]}^{y}}=\exp\left(\frac{-\Delta G{}^{\circ}}{RT}\right).$$

Since in this section the activity of water in the solution is assumed to be essentially 1 and the activity of pure solid phase is also unity, Eq. (10) is simplified further as

(11) $$P_{eq}{\left(H_{2}\right)}^{y}=\exp\left(\frac{-\Delta G{}^{\circ}}{RT}\right).$$

Taking the log (base 10) of this relationship yields

(12) $$\log\left\{P_{eq}\left(H_{2}\right)\right\}=\frac{-\Delta G{}^{\circ}}{yRT\ln(10)}.$$

Table 1 summarizes the results of the calculations using these equations to determine the equilibrium fugacity, *P_eq_*(H_2_), for the reaction of Nb with water in dilute aqueous solutions where the activity of water can be estimated as unity.

It is interesting to note that since Nb does not form soluble ions in water at any pH between 0 and 14, that Eq. (8) holds for any reaction with an aqueous solution. This means that the fugacity of hydrogen for the reaction of Eq. (7) holds for all pHs and that the hydrogen fugacity is not a function of pH as it is for metals that form soluble ions at low pH values [36].

### B. Reactions With Water Vapor

The hydrogen fugacity that can be generated on the surface of bare Nb by reaction with water vapor in air, inert gases, or vacuum is the next issue of concern. Since relative humidity (RH) is the ratio of the activity of water vapor to that for equilibrium with pure liquid phase water, RH is also the activity of water for use in calculation of hydrogen fugacity as in Eq. (10). Substituting RH for the activity of water in Eq. (10) and solving for hydrogen fugacity as in Eq. (12) yields

(13) $$\log\left\{P_{eq}(H_{2})\right\}=\frac{-\Delta G{}^{\circ}}{yRT\ln(10)}+\log(RH).$$

This relationship is very interesting as one can readily see that if one were to conduct an experiment in H_2_(g) at a moderate pressures, the activity of hydrogen in the sample may actually be greater than that corresponding to the gas pressure if significant amounts of water vapor are present in the gas. For example, CaSO_4_ desiccant keeps the relative humidity in a desiccator below an estimated 0.01 % RH. Substituting this value into Eq. (13) using Table 1 indicates that the equilibrium fugacity for this environment is still greater than 10^21^ Pa (10^16^ bar). Of course, mass transport will limit the reaction rate kinetics compared to the kinetics of absorption or desorption such that the steady state values will be well below this theoretical limit, but this calculation clearly shows that, at least theoretically, exposure to moderate pressures of water vapor can result in more hydrogen absorption by Nb than exposure to high pressure hydrogen gas. In practice, passivation of the surface limits hydrogen absorption, but significant hydrogen absorption can occur in nominally inert environments if water vapor is present and the surface cannot passivate.

### C. Solvents and Cutting Fluids

In the case of organic solvents and cutting fluids, the moisture content of these fluids will contribute to hydrogen uptake in the same manner as water vapor impurities in air, inert gases, and vacuums. That is, it will occur driven by the fugacity given by Eq. (13). Typically, these fluids are left open to the air or stored in partially sealed containers and will have sufficient time for the activity of the water in these fluids to reach equilibrium with the water vapor in the laboratory air. Drying these fluids and keeping them dry may be a means for processing Nb with a minimum of hydrogen uptake. A water-free electropolishing solution has been reported for Nb [49].

### D. Concentrated Acid Solutions (BCP)

The chemical etchant used to polish Nb that is commonly referred to as BCP (for buffered chemical polish) is a 1:1:1 or 1:1:2 mixture of HF:HNO_3_:H_3_PO_4_. Assuming that this solution is mixed up from standard reagent grade chemicals where the reagent grade acids contain significant amounts of water, then the expected composition of the actual solutions is given in Table 2.

Concentrated mineral acid mixtures of this type are commonly used for polishing, etching and cleaning of metals. In many cases, HCl is used in place of HF and sometimes H_2_SO_4_ is used in place of H_3_PO_4_. The HF acid is used to provide the fluoride ion because this ion tends to form soluble complexes with Nb. That is, being more electronegative than oxygen and forming soluble species with Nb the fluoride ion tends to react with the oxide on the surface of the Nb to form soluble species in reactions of the form.

(14) $$\begin{array}{c} Nb_{2}O_{5}+(z)HF↔\left(\frac{2}{x}\right)Nb_{x}O_{y}F_{z}^{\left(5x-2y-z\right)}\\ +(5-y)H_{2}O+(2y)H^{+} \end{array}$$

where *x*, *y*, and *z* are variables that depend on the stoichiometry of the soluble ions formed by the oxide dissolution reaction. Apparently, the Nb-fluoride ion system is very complex and a book was recently published on the fluoride compounds formed with Ta and Nb [50].

Without better information on these reactions, one needs to make some assumption on the reactions and activities of reactants and products. Palmieri et al., [49] reported that the reaction sequence during BCP is: (i) reaction with HNO_3_ to form niobium oxide:

(15) $$6Nb+10HNO_{3}↔3Nb_{2}O_{5}+10NO+5H_{2}O$$

followed by (ii) the dissolution of the oxide by reaction with HF

(16) $$Nb_{2}O_{5}+10HF↔2NbF_{5}+5H_{2}O$$

making the total reaction:

(17) $$3Nb+5HNO_{3}+15HF↔3NbF_{5}+5NO+10H_{2}O.$$

The literature indicates that the product of this reaction, NbF_5_, is unstable in the presence of water spontaneously decomposing to form HF and Nb oxide [26]. However, the goal of the current exercise is to make an estimate of the thermodynamic limit for the fugacity of hydrogen that will drive hydrogen absorption in the BCP environment. There are two approaches that can be taken for this calculation. First, one can assume a reaction of the form

(18) $$xNb(s)+yH_{z}Ac↔Nb_{x}Ac_{y}(cx)+(yz)H_{2}(g)$$

dominates the production of hydrogen and then calculate the thermodynamic limit for the hydrogen fugacity for this reaction in this environment. For BCP, the literature indicates that HF is the acid molecule most likely to react with Nb in this manner and one possibility for stoichiometry of this reaction is

(19) $$2Nb(s)+10HF↔2NbF_{5}+5H_{2}(g)$$

the change in Gibbs free energy for this reaction with all reactants in their standards states (ΔG°) is then

(20) $$\begin{array}{c} \Delta G{}^{\circ}=\left\{2*G(NbF_{5})+G(H_{2})\right\}\\ \;-\left\{2*G(Nb)+10*G(HF)\right\} \end{array}$$

or

(21) $$\begin{array}{c} \Delta G{}^{\circ}=\left\{2*(-1698.0)+0\right\}\\ \;-\left\{0+10*(-296.82)\right\}\\ \;=-427.8kJ/mol. \end{array}$$

The constant K for this reaction at equilibrium is

(22) $$K_{eq}=\frac{{\left[NbF_{5}\right]}^{2}{\left[P\left(H_{2}\right)\right]}^{5}}{{\left[Nb\right]}^{2}{\left[HF\right]}^{10}}=\exp\left(\frac{-\Delta G{}^{\circ}}{RT}\right).$$

Taking the base 10 logs of this relation assuming the activity of the metal is unity yields

(23) $$\log\left[P(H_{2})\right]=\left(\frac{-\Delta G{}^{\circ}}{5RT\ln(10)}\right)+2\log[HF].$$

Assuming that the concentration of HF in the acid is a good estimate of its activity, then Eq. (23) becomes

(24) $$\log\left[P(H_{2})\right]=14.99+1.96\approx 16$$

indicating a hydrogen fugacity of ≈10^16^ bar (10^21^ Pa). This fugacity appears low compared to that that can be expected due to the reaction of Nb with the water brought into this solution from the reagent grade acids shown in Table 2. If one estimated the activity of water in this solution by the ratio of the molar concentration of water in this solution to that of pure water, then one gets an estimated activity of 0.423 for water in BCP 1:1:1 and 0.378 for water in BCP 1:1:2. Substituting these into Eq. (10) for the formation of the pentoxide gives

(25) $$\log\left\{P_{eq}(H_{2})\right\}=20.33+\log[H_{2}O]\approx 19.9.$$

This then indicates that the fugacity driving hydrogen absorption may be as high as ≈10^20^ bar (10^25^ Pa).

### E. Electropolishing

Niobium is frequently electropolished (EP) in a solution of H_2_SO_4_ and HF with a 9:1 volume ratio at current densities in the range 50–100 mA/cm^2^. As with the chemical polishing solutions, this solution will contain water and Table 3 contains the information estimated for the actual composition of this solution.

The thermodynamic limit for the hydrogen fugacity in this solution in the absence of any applied currents can be estimated in the same fashion as that used for the chemical polishing solutions. That is, direct reaction with HF to produce NbF_5_ according to the reaction of Eq. (19) can be expected to produce hydrogen fugacities up to

(26) $$\log\left[P(H_{2})\right]=14.99+2\log[HF]\approx 16$$

and the reaction with the water in the solution can be expected to produce hydrogen with fugacities up to

(27) $$\log\left\{P_{eq}(H_{2})\right\}=20.33+\log[H_{2}O]\approx 19.4.$$

Direct chemical reaction in the absence of polarizing current densities will produce hydrogen at fugacities up to 10^16^ bar (10^21^ Pa) according to Eq. (26) and 10^19.4^ bar (10^24.4^ Pa) according to Eq. (27).

For electropolishing, an auxiliary electrode is added to the solution and a power supply is used to apply a current (or potential) to the sample that stimulates the polishing reactions. The applied current raises the potential of the sample stimulating anodic dissolution (oxidation) reactions and suppressing cathodic (reduction) reactions. Under ideal conditions, only anodic reactions occur on the sample (anode) and only reduction reactions occur on the auxiliary electrode that is now the cathode. However, in the case of an active metal such as Nb in an aqueous electrolyte, the overpotential for the dissolution reactions is so great that it is difficult to completely suppress cathodic reactions on the anodic sample. In the case we are examining here, the anodic reactions would be either Nb dissolution

(28) $$Nb↔Nb^{+5}+5e^{-}$$

or repassivation according to a reaction of the form

(29) $$2Nb+5H_{2}O↔Nb_{2}O_{5}+10H^{+}.$$

Normally, Nb does not form soluble ions in aqueous solutions, but with the fluoride ion present soluble complexes form that reduce the thickness of passivating films accelerating dissolution reactons. The cathodic reaction in this solution is then

(30) $$2H^{+}+2e^{-}↔H_{2}(g).$$

Since this solution no longer contains the HNO_3_ molecule, the reduction reaction

(31) $$3H^{+}+3e^{-}+HNO_{3}↔NO+2H_{2}O$$

that is included in Eq. (17) cannot occur in this solution. This is why the addition of nitric acid to the BCP solution accelerates the polishing reactions. The question to be addressed here is to estimate the extent that the applied anodic current suppresses the fugacity of hydrogen on the sample surface during electropolishing. If one assumes normal Tafel kinetics for the reaction of Eq. (26) on the surface of Nb, then the reaction rate measured by the current (*i_c_*) is related to the overpotential driving the reaction (*η*) according to an equation of the form

(32) $$i_{c}=i_{0}\exp\left(\frac{-(1-\alpha )nF\eta }{RT}\right)$$

where *n* = 1, *F* is Faradays constant, *α* is the reaction symmetry constant (typically 0.5), and *i*_0_ is the exchange current density. Rearranging and combining terms Eq. (32) becomes

(33) $$\eta =\beta \log\left(\frac{i_{c}}{i_{0}}\right)$$

where *β* is the Tafel slope. The Tafel slope (*β*) and the exchange current density (*i*_0_) are constants that depend on the material and the solution. Tabulated values for these constants can be found in the literature, but data is not available for bare Nb metal in the EP solution. However, if one assumes that the values reported for a 1.0 mol/L HCl solution are a good estimate for those that will be observed in the EP solution, then one can make estimates of the effect of current densities of the magnitude used in electropolishing on the hydrogen fugacity driving hydrogen absorption. It is reported that in this environment the values for *β* and *i*_0_ are 0.10 (V) and 1 × 10^−4^ (mA/cm^2^) respectively [4, 51]. Assuming that the EP current density is 100 mA/cm^2^, then Eq. (33) becomes

(34) $$\eta =(0.10)\log\left(\frac{100}{0.0001}\right)\approx 0.6.$$

That is, the applied current density corresponds to the application of an overpotential of approximately 0.6 V. Since a simple model for the reversible transfer of ions between electrodes will show that

(35) ΔG=−nFE.

This illustrates the fact that one could just as easily done the calculations in this document in terms of potentials as well as free energies. Substituting Eq. (31) into Eq. (8) yields

(36) $$\log\left\{P_{eq}(H_{2})\right\}=\frac{2FE}{RT\ln(10)}=\frac{\eta }{0.0296}\approx 20.3.$$

That is, it is estimated, assuming that the Tafel slope and exchange current density in the EP solution are similar to those for Nb in 1.0 mol/L HCl, that the application of an anodic current of 100 mA/cm^2^ will lower the fugacity driving hydrogen absorption by 20 orders of magnitude. Since Eq. (27) found that the expected fugacity for unpolarized conditions was less than this value (19.4 maximum estimate), this estimate indicates that the hydrogen fugacity for this condition is less than

(37) $$\log\left\{P_{eq}(H_{2})\right\}\leq -1.$$

Of course, this estimate contains a large number of rough approximations one of which is the assumption that the applied current is evenly distributed so that local cell actions cannot occur in occluded regions of the surface, but it is interesting to find that the empirically determined polishing conditions that give the best results are almost exactly those that this estimate indicates would suppress H_2_ bubbling from the surface. This could be an indication that suppression of H_2_ bubbling helps one obtain a better surface finish.

## 4. Discussion

### A. Thermodynamic Analysis

The analysis presented in this document is based entirely on the principles of thermodynamics and the assumption of equilibrium. When reactions are occurring and currents are flowing, actual conditions will deviate from those of a reversible equilibrium and the magnitude of this deviation increases with increasing reaction rate or current density. For this analysis, all factors in the system were identified and assumed constant except for the hydrogen pressure or fugacity. Then, it was assumed that all of the excess energy driving the reaction forward was converted reversibly into hydrogen pressure. The reversible equilibrium fugacity calculated in this manner represents the upper limit that the system is attempting to reach for these boundary conditions.

Since the fugacities determined are based on the assumption of equilibrium, they can only be achieved under fully reversible conditions. Irreversible losses will increase with the deviation from equilibrium conditions. This creates a discrepancy between the calculated theoretical hydrogen fugacity and the actual, steady state, hydrogen fugacity that the metal will experience. This discrepancy will increase with the magnitude of the theoretical value. However, if one assumes activation controlled kinetics and that the environments do not contain species that dramatically alter the kinetics of the relevant processes, then one can expect greater hydrogen absorption under conditions with a higher equilibrium hydrogen fugacity. In other words, the equilibrium fugacity quantifies the thermodynamic forces behind hydrogen absorption and evolution or the natural propensity for these processes on Nb in these environments even if the values determine are never actually achieved in practice.

### B. Kinetic Limitations

Most of the fugacities calculated for the reactions above are absurdly high and kinetics will keep these systems from reaching these theoretical values. The kinetic limit to the hydrogen fugacity is determined by the sequence of reactions required to remove hydrogen atoms from the surface as their concentration increases toward this theoretical value or upper limit. These reactions have been studied in detail on a number of different metals and typically the recombination reaction, Eq. (5), is found to be the rate determining step (in the case of active metals such as Nb, the passive film is frequently in place during these measurements making them of little value to this discussion) [51]. This implies that the fugacity on the surface may be orders of magnitude greater than the ambient pressure, but also orders of magnitude below the calculated theoretical fugacities.

Assuming that mass transport and recombination are much slower than surface adsorption and electron tunneling, then the highest hydrogen fugacities are probably reached in first milliseconds after bare metal comes into contact with the environment. Assuming that repassivation of the surface starts immediately after the initial adsorption and hydrogen reduction transient, the reformed passivating film may block hydrogen egress resulting in the hydrogen diffusion into the metal (e.g., repassivation of Al alloys starts within the first millisecond of exposure) [48]. If this is the case, then periodic rupture and repair of the passivating layer in an environment containing water could result in significant hydrogen absorption. This analysis indicates that it is theoretically possible for abrasion and periodic passive film rupture by chemical or mechanical means to introduce more hydrogen into Nb than exposure to high pressure hydrogen gas especially if one does not employ measures to remove the passive film and stimulate dissociation of the hydrogen gas for the gas phase exposures [52]. In addition, this analysis indicates that vacuum annealing to remove hydrogen could actually result in increased hydrogen levels if the sample is exposed to water or humid air before the passive film has fully reformed and passivated the surface.

### C. Electrochemical Measurements

**Fig. 6:**
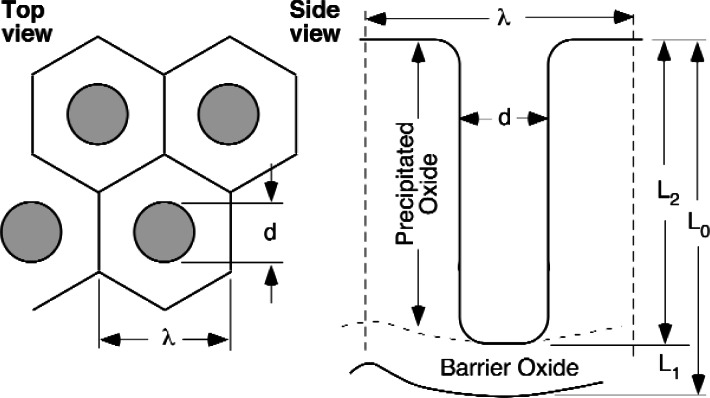
Schematic of the pore structure found on anodically polarized Al alloys [60].

A number of investigators have conducted experiments on BCP and EP processes used in the fabrication of SRF cavities. Many of these rely on empirical cell voltage measurements. While cell voltage is a reliable means for reproducing polishing conditions, it is highly dependent on the cell geometry, solution chemistry, and experimental technique. More importantly, it provides no useable information on the actual thermodynamic driving forces governing reactions on the surface of the sample. One of the most thorough studies was that of Nb in the EP environment of Tian et al., [53]. These authors used a reference electrode that has a fixed potential with respect to the hydrogen electrode that enables them to measure and control the potential difference that determines the thermodynamic forces driving the reactions (Eq. (3)). These authors found very broad current density plateaus in their current-voltage curves. The current density of the plateaus was found to depend on the concentration of the species in the environment that reacts with the Nb in the passivating oxide to form soluble complexes (the fluoride ion). These results were found to be consistent with the duplex salt film model of Landolt and co-workers [54–58]. This salt film model is very similar to that proposed in 1953 by Keller et al., [59] to explain the structure of the oxide film observed on Al alloys following anodic polarization and exploited by Miller et al., [60–64] in the development of a patented process for the fabrication of nanodot or quantum dot arrays [65]. Due to the active nature of Nb (Fig. 2), it is highly unlikely that the salt film on the surface of this metal during electropolishing is anything other than niobium oxides. However, the barrier layer at the bottom of the pores may be the sub-oxides rather than the pentoxide which would be the more stable phase in the bulk environment. The formation of niobium fluoride complexes would occur at the bottom of these pores and mass transport of the negatively charged fluoride ions to the bottom of the pores and/or the transport of the positively charged niobium-fluoride complexes out of the pores would be rate limiting (Fig. 6). These rate-limiting processes are responsible for the current density plateaus. That is, increasing the driving force has no impact on the observed rate since the boundary conditions determine the rates of mass transport are not altered. The current density generated from the area outside the pores can be approximated as zero making the total current density a function of the pore geometry and the rate of mass transport in the pore by the rate limiting process.

The issue with respect to this report is how does the application of these polarizing potentials or currents influence the propensity of Nb to absorb hydrogen from this environment and do the measurements of Tian et al., [53] demonstrate that hydrogen will not be absorbed? On a purely theoretical basis, this question is answered by the calculations in the electropolishing section above and the measurements of Tian et al. [53] support the theoretical result that indicates hydrogen absorption by the metal will be suppressed by conditions of this process. However, in practice it is difficult to control the distribution of potentials and currents on the surface of a sample in an environment where it is this far from equilibrium. In the case of Nb or Al where the bare surface current density may be orders of magnitude greater than the externally imposed anodic current [48], the surface will still be covered with oxide even if it is discontinuous and inhomogeneous. Electropolishing occurs because a steady state is reached where the oxide is removed and replaced either on opposite sides of thin homogeneous oxide film or in a complex duplex film and pore structure as shown in Fig. 6. The literature indicates that the latter is preferred at the higher current densities that provide reasonable polishing rates.

In either case, the chemical potentials of the Nb atoms in the metal are unaltered by the application of these polarizing currents and the voltage gradients they produce. The thermodynamics of the electrochemical reactions are altered because the ions participating in these reactions must move in the voltage gradient and this alters the energy changes the reactions produce. If a small region of the surface becomes shielded from the polarizing current and the voltage gradient it produces, then the thermodynamic conditions in this occluded region revert to those that exist in the absence of the polarizing currents. For example, if the mouth of the pore shown in Fig. 6 was suddenly blocked by the precipitation of niobium oxide (by hydrolysis of the Nb-F complexes) or a gas bubble, then the voltage gradient would be virtually removed allowing direct reaction between the solution at the tip of the pore and the Nb metal. According to the calculations above, this should result in the production of hydrogen gas the expansions of which would clear the blockage. Investigators have collected the gas observed during anodic polarization of metals. For example, Bargeron and Benson [66] analyzed the gas evolved from Al during anodic polarization at potentials where thermodynamic consideration would predict the gas should be oxygen and they found the gas evolved to be essentially pure hydrogen. Similar results have been reported for the gases evolved during anodization of Al alloys [4].

### D. Niobium Hydrides

The hydrogen activities calculated in terms of equivalent pressure or fugacity are clearly much greater than atmospheric pressure and hydrogen gas bubble nucleation can be expected when the surface remains bare for any significant length of time in these environments. However, recombination and bubble nucleation can be very slow compared to electron tunneling, reduction, and even mass transport. Recombination is typically found to be slower than bulk diffusion in hydrogen gas phase desorption experiments on Nb where H_2_ molecules can desorb directly without having to accumulate and nucleate bubbles [67, 68]. Therefore, while the actual activity of hydrogen on the surface may be considerably less than that estimated by these equilibrium calculations, it will be considerably greater than the external pressure and very high hydrogen activities may be reached in the first few moments after water contacts bare metal because electron tunneling will initially generate adsorbed hydrogen very quickly compared to the time required for mass transport or other reactions. The logical conclusion is that while the hydrogen activity will not reach the theoretical values, it will be large and this will result in a large driving force for hydrogen absorption and reactions with hydrogen including the formation of niobium hydrides.

**Fig. 7:**
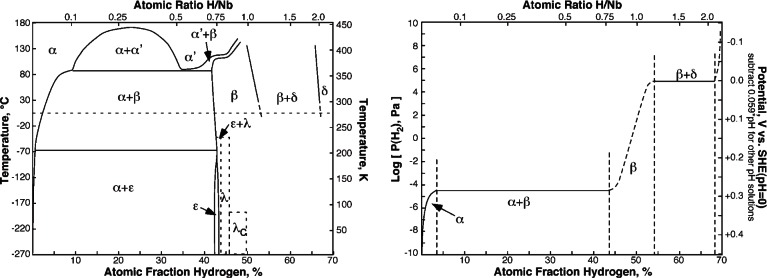
Niobium-Hydrogen phase diagram reported by Manchester and Pitre [69] (a) and 25 °C equilibrium pressure and potential estimates for hydride phases based on the data of Fromm and Jehn [71] for the α and α + β regions and Manchester and Pitre [69] for the β + δ region (b).

Niobium hydrides have been studied extensively and a number of different hydride phases have been identified [69]. However, the niobium-hydrogen phase diagram has not been completely determined and there are a number of phases that authors have identified by one technique or another that have not been confirmed or the phase field clearly identified. Manchester and Pitre [69, 70] reviewed the literature on niobium-hydrogen phases and compiled the data into the assessment of the temperature-composition diagram shown in Fig. 7(a). This diagram shows 7 phases:

1. *α – Phase*: The phase is essentially the pure Nb body centered cubic (bcc) phase interstitially alloyed with H atoms randomly distributed over tetrahedral sites in the crystal lattice [69].
2. *α′ – Phase*: The α′ phase is a random interstitial alloy of H in bcc Nb with essentially the same crystal structure as the α phase. This designation is used for H concentrations above about 24 % mole fraction [67–69].
3. *β – Phase*: The phase has a face centered cubic (fcc) orthorhombic structure with H atoms occupying four interstitial sites per unit cell in an ordered arrangement with essentially the stoichiometry of NbH. This phase forms below 150 °C, but undergoes a series of ordering transformations below 30 °C to 70 °C resulting in the and phases.
4. *δ – Phase*: The phase has the fluorite structure in which the Nb atoms have an fcc arrangement with the H atoms occupying tetrahedral sites. The boundaries of this phase field are poorly known and dashed lines are used in the diagram of Fig. 7(a) to indicate this fact.
5. *ε – Phase*: The β phase transforms into the ε phase at temperatures below −69 °C. This phase has a narrow concentration band around the stoichiometry of Nb_4_H_3_ and forms out of the β phase by long-range ordering the H atoms in the tetrahedral sites while the Nb atoms remain unaffected. Apparently, H has significant mobility at these low temperatures and Manchester and Pitre [69] report that studies have observed H motion at temperatures as low as 0.05 K.
6.,7. *λ and λ_c_ Phases*: Manchester and Pitre [69] report that at least 5 other phases have been reported in the literature, but the experimental evidence is uncertain or unconfirmed. These phases are discussed in their paper, but with the exception of the *λ* and *λ_c_* phases, these authors elected not to include these phases on the temperature-composition diagram. The phase field for these phases is poorly known and dashed lines are used in the diagram of Fig. 7(a) to indicate this fact.

The diagram of Fig. 7(b) was determined using the solubility data of Fromm and Jehn [71] for the α phase field and α + β two phase field. The thermodynamic data reported by Manchester and Pitre [69] was used to calculate the equilibrium hydrogen pressure over the β + δ two phase region. No data was found on the solubility of H in either the β phase or the δ phase and dashed lines are used in this diagram to illustrate this uncertainty. This diagram shows the pressure or fugacity of hydrogen that is in equilibrium with the phase of the specified composition at 25 °C. Theoretically, a constant H activity should result in the two phase regions where the ratio of the phases changes with the overall composition, but not H activity. Added to this diagram to make this relevant to the calculations above is a potential scale where the electrode potential on the standard hydrogen electrode (SHE) scale for a solution of pH = 0 that would generate the same H fugacity is calculated. For solutions of other pH, one would subtract 0.0593 V/pH unit from the value on this scale keeping in mind that the direction of this scale is inverted to agree with the pressure axis. This diagram shows that an H fugacity of slightly less than atmospheric pressure (7.0 × 10^4^ Pa) is all that is required to nucleate the most H rich niobium hydride phase. Assuming that an H concentration gradient forms with a decreasing concentration from the surface into the interior of the metal, then one could expect that all three of the room temperature phases may form for this postulated condition (α, β, and δ). If the metal is not treated to remove H or dissolve these phases into the bulk, then these phases will be present when the temperature is reduced to superconducting temperatures and the ordering reactions will result in the formation of the low temperature phases (ε, λ, and λ_c_).

## 5. Conclusions

The purpose of this report was to evaluate the feasibility of the hypothesis that hydrogen may be absorbed during processing of Nb and alter the performance of this metal during subsequent processing steps (e.g., forming) or in service. The goal of this evaluation is to enable better interpretation of the effects of changes in processing conditions on the performance of accelerator cavities or the development of new or modified processes that optimize the performance of Nb SRF cavities. This evaluation consisted of (i) identification of the steps, conditions, and required reactions, (ii) examination of the scientific literature for established precedent in other metals and alloys, (iii) thermodynamic assessment of feasibility of the reactions with quantification of the maximum possible hydrogen activity, and (iv) examination of the assumptions and limitations of the analysis. This examination found that the hypothesis of hydrogen absorption during SRF processing is consistent with published scientific literature and thermodynamic principles.

This investigation found that not only is hydrogen evolution and absorption feasible when water comes into physical contact with bare (unpassivated) Nb metal, but that the driving forces for these reaction are quite large. These large driving forces indicate that whenever water comes into direct physical contact with bare Nb metal, the reactions will be very rapid until reaction products block mass transport and limit the access of the reactants to each other (ie. passivation). Since the tunneling of electrons to the adsorbed hydrogen cations of the water molecule will precede the reaction between the oxygen and niobium to form the oxide, hydrogen reduction and absorption appears to be inevitable under these conditions. The quantity actually absorbed will vary with the relative rates and environmental conditions. Also, desorption may occur in some cases while in others a more continuous and impervious passivating oxide may block desorption. All of the aqueous environments examined have the capability of producing hydrogen when they react with bare Nb metal and the thermodynamic calculations indicate that these reactions are capable of producing hydrogen on Nb at activities greater than those one can obtain from exposing this metal to high-pressure hydrogen gas. Therefore, while reaction kinetics may limit the actual quantities generated and absorbed, the possibility of hydrogen absorption should always be considered with a process that ruptures the passivating oxide on this metal in the presence of water molecules.

Theoretically, polarizing currents can be applied to suppress hydrogen evolution. However, the distribution of this current over the surface of the sample would be a critical factor in determining the success of this approach. Natural perturbations to the current distribution or the local shielding of occluded regions from the polarizing current could result in the establishment of local electrochemical cells where hydrogen evolution is favorable. This is a significant possibility in systems that are far from equilibrium with a large driving force for reaction between the metal and the environment. The literature indicates that hydrogen evolution can occur on Al alloys even when large anodic currents are applied. Therefore, while theoretically it is possible to prevent hydrogen uptake with anodic polarization, in practice it would be unreliable particularly for active metals where conditions are far from equilibrium and post-processing out-gassing treatments and controlled passivation are probably the best approaches for the elimination of hydrogen.

## Figures and Tables

**Table 1:** Gibbs free energy, equilibrium potential (E_eq_), and equilibrium hydrogen fugacity (P_eq_(H_2_)) for reactions between Nb and water for the thermodynamic data of Wagman et al., [35]

Product	G_f_(Nb_x_O_y_)	X	Y	n	DG° kJ/mol	E(eq) V vs. SHE	log (P(H_2_)) bar	log (P(H_2_)) Pa
NbO	−378.6	1	1	2	−141.47	−0.733	24.78	29.78
NbO_2_	−740.5	1	2	4	−266.24	−0.690	23.32	28.32
Nb_2_O_5_	−1766.0	2	5	10	−580.35	−0.601	20.33	25.33

**Table 2:** Estimated Composition of BCP solutions

Acid	MW, g/mol	Reagent Conc, mass %	BCP 1:1:1 mol/L	BCP 1:1:1 mass %	BCP 1:1:2 mol/L	BCP 1:1:2, mass %
HF	20.01	49.0	9.63	13.4	7.23	9.6
HNO_3_	63.01	70.4	5.30	23.3	3.98	16.7
H_3_PO_4_	98.00	85.5	4.93	33.7	7.40	48.4
H_2_O	18.02	100	23.48	29.5	21.02	25.3

**Table 3:** Electropolishing (EP) Solution Composition

Acid	MW, g/mol	Reagent Conc, mass fraction (%)	EP 9:1 mol/L	EP 9:1, mass fraction (%)
HF	20.01	0.490	2.89	0.033
H_2_SO_4_	98.08	0.960	16.20	0.896
H_2_O	18.02	1.000	7.02	0.071

## Footnotes

1 The author may have presented varying points of view to help the reader consider and evaluate scientific issues. These should not be considered the conclusive opinion of the author nor that of NIST. Only thoroughly reviewed and confirmed results should be used for critical decision making. The identification of any commercial product or trade name does not imply endorsement or recommendation by the author or NIST.
